# Advantages of Array-Based Technologies for Pre-Emptive Pharmacogenomics Testing

**DOI:** 10.3390/microarrays5020012

**Published:** 2016-05-28

**Authors:** Al Shahandeh, Daniel M. Johnstone, Joshua R. Atkins, Jean-Marie Sontag, Moones Heidari, Nilofar Daneshi, Elvis Freeman-Acquah, Elizabeth A. Milward

**Affiliations:** 1 School of Biomedical Sciences and Pharmacy, The University of Newcastle, Callaghan 2308, Australia; Ali.Shahandeh@uon.edu.au (A.S.); Joshua.Atkins@uon.edu.au (J.R.A.); Jean-Marie.Sontag@newcastle.edu.au (J.-M.S.); Moones.Heidari@uon.edu.au (M.H.); Nilofar.Daneshi@uon.edu.au (N.D.); 2 The Bosch Institute and Discipline of Physiology, University of Sydney, Sydney 2006, Australia; Daniel.Johnstone@sydney.edu.au; 3 Institute of Genetics and Cytology, School of Life Sciences, Northeast Normal University, Changchun 130024, China; davfreeman82@yahoo.com

**Keywords:** microarray, next-generation sequencing, pharmacogenomics, personalized healthcare

## Abstract

As recognised by the National Institutes of Health (NIH) Precision Medicine Initiative (PMI), microarray technology currently provides a rapid, inexpensive means of identifying large numbers of known genomic variants or gene transcripts in experimental and clinical settings. However new generation sequencing techniques are now being introduced in many clinical genetic contexts, particularly where novel mutations are involved. While these methods can be valuable for screening a restricted set of genes for known or novel mutations, implementation of whole genome sequencing in clinical practice continues to present challenges. Even very accurate high-throughput methods with small error rates can generate large numbers of false negative or false positive errors due to the high numbers of simultaneous readings. Additional validation is likely to be required for safe use of any such methods in clinical settings. Custom-designed arrays can offer advantages for screening for common, known mutations and, in this context, may currently be better suited for accredited, quality-controlled clinical genetic screening services, as illustrated by their successful application in several large-scale pre-emptive pharmacogenomics programs now underway. Excessive, inappropriate use of next-generation sequencing may waste scarce research funds and other resources. Microarrays presently remain the technology of choice in applications that require fast, cost-effective genome-wide screening of variants of known importance, particularly for large sample sizes. This commentary considers some of the applications where microarrays continue to offer advantages over next-generation sequencing technologies.

## 1. Introduction

Many reviews cover the advantages of emerging genome-scale sequencing technologies in diverse contexts and these will not be revisited here. Yet, in the rush to adopt these promising new technologies, researchers and funding bodies sometimes fail to recognize that, in some contexts, the use of these platforms is unjustifiable and that high-density genotyping arrays continue to be a far more appropriate choice. Fortunately, since these sequencing technologies are frequently still very costly, there is increasing awareness that the newer approaches are not always better.

For example, the practical advantages of high-density genotyping arrays over genome-scale sequencing in studies involving large numbers of samples have been acknowledged in the lead-up to implementation of President Obama’s PMI. This is summarized in the September 2015 report of the PMI Working Group to the Advisory Committee to the Director of the US NIH [1]. The Working Group noted that, in most circumstances, issues with the cost, imperfect results and expectation of technology obsolescence made genome-scale sequencing approaches presently inappropriate for large numbers of individuals. The Working Group recommends ongoing monitoring to assess when the balance of the scientific value over the costs and capabilities of such methods reaches a “tipping point”. Meanwhile, as recognized by the Committee, reasonable utility can be achieved at an affordable cost by high-density genome-wide arrays testing common and rare gene variants.

As addressed elsewhere in the Special Issue on “Microarrays in the Era of Next Generation Sequencing”, microarrays continue to be used in diverse applications. Examples include detection of chromosomal abnormalities in cytogenetics using array-based comparative genome hybridization, in providing rapid turnaround for prenatal investigations using limited amounts of DNA and in replacing other technologies such as fluorescence *in situ* hybridization (FISH) for some oncology applications. In the present article we will focus primarily on the area of pharmacogenomics, where arrays are now being widely used both in basic research and in research into the effective translation of pharmacogenomics into clinical practice.

While microarrays do not enable new gene variants to be discovered and are, therefore, generally not well-suited to clinical genetics applications seeking to identify novel disease-associated variants, high throughput genome-wide array technology can still provide the capacity to simultaneously assess essentially all single nucleotide polymorphisms (SNPs) of known functional importance in the human genome [2,3,4,5,6]. This level of genomic coverage is sufficient for many current applications in medicine and research, including most pharmacogenomics applications, as will be discussed in more detail below. The rapid output, affordability, and availability of microarray technology, along with its high accuracy and established and validated pipelines for data analysis and variant calling, make it the logical choice for such applications [7].

This is particularly true for large sample sizes, for example in large genome wide association studies (GWAS), where microarrays have been, and in most instances continue to be, the only economically viable option. Using microarrays, scientists from around the globe can contribute data of various kinds (including genomic, epigenetic, and transcriptomic data) to massive consortium project, even when only able to afford to study a small number of samples. Although sample size restricts the capacity to detect association in small studies, analyses of collective pooled sample sets can be extremely powerful. Hence, while next-generation sequencing (NGS) technologies are essential for discovery-driven research focused on the identification of novel sequences, it may often be unnecessary and even potentially a fiscally irresponsible misuse of research funding [7] to use such methods for profiling common variants (e.g., SNPs) across a large number of patients or in a variety of other applications where detecting novel sequences is not the primary goal.

Although less relevant in the context of this article, array technology is not only still useful for genomic studies but also continues to offer many advantages for various other kinds of high-throughput studies, including transcriptomics, where it remains the platform of choice for many studies. For example, in 2014, RNA-seq data was uploaded into the Gene Expression Omnibus (GEO) database for around 9000 samples whereas microarray data was uploaded for over 54,000 samples [8]. Microarray-based clinical tests provide a powerful tool for simultaneous measurement of the relative expression levels of a large number of well-established clinically relevant genes in the context of disease or drug responses. There is a wide range of applications for gene expression microarrays in providing RNA profiles associated with different disease states for various purposes, including monitoring pharmacological responses in clinical trial participants and identifying suitable drug treatments for individual patients, as reviewed elsewhere [9,10,11].

In view of such considerations, the relatively high costs of sequencing often appear hard to justify in a climate where increasing numbers of researchers are losing funding. Even ignoring the often higher cost of consumables and equipment for NGS as compared to microarray, the greatest cost often lies in the labour. The cost of next-generation whole-genome and transcriptome sequencing is dropping rapidly, and may one day match the cost of microarray-based methods. However the frequent claims of the $1000 genome or even of costs comparable to those of arrays usually do not adequately take into account the cost of time and human resources in sample preparation, sequence alignment, and filtering through huge volumes of data to catalogue SNPs or other information of interest, let alone the infrastructure required for sequencing, data processing, and storage [7]. Microarrays, therefore, continue to provide a highly cost-effective choice in contexts involving samples from relatively large groups of individuals, such as pharmacogenomics. This review will primarily consider the enduring value of microarrays in pharmacogenomics. We will, first, very briefly review the current status of pharmacogenomics in clinical practice before going on to consider criteria that a genotyping platform will need to meet to be relevant to clinical pharmacogenomics in the future. We will consider how microarrays measure up to these criteria and briefly discuss some examples of successful applications of microarray in research into the effective translation of pharmacogenomics into clinical practice.

## 2. Pharmacogenomics in Practice

As defined by the Food and Drug Administration (FDA), pharmacogenomics studies variations of DNA (genomic) and RNA (transcriptomic) characteristics as related to drug response, providing information which can be used to inform appropriate drug selection or dosage regimens for individual patients [12]. This relies on the identification of SNPs and other variants in genes known to be important in pharmacokinetics or pharmacodynamics. Considerable ongoing research focuses on identifying and profiling these variants; however, currently only a few gene variants are considered to have a firm evidence base for clinical actionability.

For most drugs, information on clinically-actionable gene variants (for which either a change of medication or a change of dose are recommended) can be obtained by screening only a small portion of the genome. The guidelines of the Clinical Pharmacogenetics Implementation Consortium (CPIC), supported by the US NIH and available through the Pharmacogenomics Knowledge Base (PharmGKB) [13], list only 17 genes with “high” evidence (Level 1A or 1B) of a drug-modifying effect (see Table 1), with “moderate” evidence (Level 2A or 2B) for an additional 40 genes. While further research is likely to reveal a number of other variants that modify the pharmacokinetic or pharmacodynamics profiles of new or existing drugs, the degree of screening required is, therefore, unlikely to extend beyond the capabilities of microarray technology for some time into the future.

Several microarray-based tests that simultaneously examine variations in multiple genes are approved by the FDA and have entered practice. These include AmpliChip CYP450 from Roche and MammaPrint from Agendia. Although whole genome sequencing and whole exome sequencing of potential pharmacogenomic gene variants have been reported previously [33,34], as far as we are aware the first and, to date, the only FDA-cleared NGS platform for *in vitro* diagnostic testing is a single gene test only, specifically a cystic fibrosis mutation detection test utilizing the Illumina MiSeqDx System [35]. For these and other reasons described elsewhere in this article, microarrays are likely to continue to be relevant and beneficial for clinical practice for some time into the future.

## 3. Minimum Criteria for a Clinically Useful Pharmacogenomics Platform

In practical terms, irrespective of the technology used, the ideal pharmacogenomics platform should meet the following minimum criteria [36,37].

### 3.1. Analytical Validity

Ideally, a pharmacogenomics test should have high analytical specificity and sensitivity, with appropriate laboratory quality assurance and assay robustness. The data generated should be highly accurate with minimal errors in calling of gene variants. However, accuracy issues continue to restrict the usefulness of NGS. Even the most advanced sequencing platforms still have a base call error rate that, although usually proportionately small compared to many other technologies, is amplified by the large number of reads performed in an NGS experiment. This can make it difficult to distinguish polymorphisms from sequencing errors [38,39,40].

Various kinds of bias affect the analytic validity of NGS data [38,41]. Systematic bias involves non-random errors arising because of inaccuracies inherent in the platform and associated protocols, including errors deriving from the methods used to generate the original sequencing library [38]. Systematic errors can also reflect coverage bias, which may occur in regions where the genome sequence, chemistry, or conformation affects data output. This form of bias can in part be platform-dependent but can also occur across platforms and the error involved can be substantial—for example, a 2012 study by Quail and colleagues [42] of three platforms, Ion Torrent Personal Genome Machine, Pacific Biosciences PacBio RS, and Illumina MiSeq, found that output from sequencing extremely AT-rich genomes contained high levels of bias and errors with no coverage of almost 30% of the genomes investigated. Another important component of systematic bias—pertinent to laboratory quality assurance and assay robustness—isbatch effects relating to external factors such as reagent variability [38,41].

Sequencing accuracy for leading longer established technologies such as Illumina is often over 99% [43,44]. For single nucleotide variants differing from the reference genotype, the error rates for whole-genome and whole-exome sequencing of Illumina HiSeq or Complete Genomics have been estimated to be up to 0.1% or 0.6%, respectively, using replicate high-coverage sequencing of human blood and saliva DNA samples [39] and advances such as the HiSeq X Ten model and the Complete Genomics Long Fragment Reads technology [45] are achieving considerably better rates.

These are relatively well-established technologies which have been in use and evolving for some time, facilitating development of expertise and optimisation of protocols. However, in general, these technologies tend to be relatively costly compared to some of the other platforms, although these are sometimes less accurate with higher error rates [38,43,46]. Sequencing accuracy of the PacBio platform has been reported to be in the range of 80%–90% [43,47], with a study comparing three important platforms—Ion Torrent Personal Genome Machine, Pacific Biosciences PacBio RS and Illumina MiSeq (reviewed in more detail in the study in question)—on a set of four microbial genomes observing error rates of below 0.4% for the Illumina platform, 1.78% for Ion Torrent and 13% for PacBio sequencing [42]. The number of error-free reads, without a single mismatch or insertion and deletion (indel), was 76.45%, 15.92%, and 0% for MiSeq, Ion Torrent, and PacBio, respectively. The PacBio errors were evenly distributed, whereas MiSeq produced more errors after long (>20-base) homopolymer tracts or for GC-rich motifs. The affordable and widely used Ion Torrent platform produced erroneous base numbers for homopolymers >8 bases long and failed to generate reads entirely for long (>14-base) homopolymer tracts, along with strand-specific errors that were not associated with any obvious motif.

The long reads and low error rates of early NGS platforms such as Roche 454 sequencers made error correction relatively unimportant [38]. Most error-correction programs have primarily addressed substitution errors, since these have been an issue for the widely used Illumina machines; however, short read platforms, such as Ion Torrent, are more prone to other sorts of errors, such as indels [48,49,50]. As approaches to error correction are refined for each emerging technology, the accuracy of the output is likely to improve considerably; one of the potentially most exciting new developments, the MinION, has a raw sequencing error rate of about 12% which can be improved to 0%–3% with hybrid or *de novo* error correction [51]. Such errors are often relatively unimportant for discovery-based applications in research settings or clinical investigations to identify disease-related mutations in families, where candidate variants can be validated using a range of other approaches. However, such errors are more of a problem in clinical pharmacogenomic contexts requiring fast and reliable decisions about medications, where microarrays and, in particular, validated custom-designed arrays for pharmacogenomics and other applications can offer more reliable options [10,34,52].

### 3.2. Clinical Validity and Utility

While, as discussed above, a test must be able to evaluate the measure of interest accurately (analytical validity), a test only has clinical validity if what is being measured correlates closely with some clinical outcome of interest. In the context of pharmacogenomics, clinical validity translates to the ability of a genomic test to detect or predict the response to a drug correctly and with high specificity and sensitivity.

Even though a test may have analytical and clinical validity, it still need not necessarily be clinically useful if, for example, the information provided by the test does not serve any useful purpose for the physician, the patient or other relevant stakeholders. The clinical utility of a genomic test, in the broadest sense, can be considered to refer to the usefulness of the information it provides in enabling clinicians, patients or other stakeholders to make appropriate health-related decisions; a comprehensive list of factors influencing clinical utility is provided by the Centres for Disease Control and Prevention (CDC) [53]. Relevant considerations include whether appropriate equipment, expertise and validated educational materials are available to allow effective use of test results in healthcare decision making. In the present context the concept of clinical utility is used primarily with regard to the requirement that a pharmacogenomics test should provide information of value to decision making by health professionals or patients. For example, the test may help decide on applicable interventional approaches.

The availability and accessibility of a test also affect the extent of its usefulness. Ideally, pharmacogenomic test results would be accessible by professionals working at point-of-care (e.g., doctors or pharmacists), using a simple database query that would link the prescribed drug with a patient’s genotype to identify any recommended modifications to the treatment or dose. In our experience, and as evidenced by large-scale pre-emptive clinical pharmacogenomics testing programs described in more detail below [54], pharmacogenomics test results can be relatively easily and rapidly extracted from DNA array data using potentially automatable procedures, and can be interpreted by personnel with relatively little training and experience.

In contrast, one of the main practical barriers to implementation of NGS in clinical practice is the relative scarcity of personnel capable of handling and interpreting NGS data. Expertise in recognizing errors from true calls (which will also reflect analytical validity) is critical in ensuring the correct prescription is received by patients, and avoiding possible claims of negligence. The interpretation of NGS data presently involves extensive and time consuming analyses that require expert human judgement. While similar considerations also apply for microarray data, at present there are relatively well-established automated algorithms and pipelines for array data processing [55], whereas NGS data analysis still more commonly requires considerable human input and judgement [46,56].

## 4. Additional Technical Issues

Speed is another concern that is often raised. The entire turnaround time, from DNA collection to reporting, should be no longer and, if possible, less than that of standard pathology tests. However, while the speed of the new sequencing technologies continues to increase, the requirements of error correction and other analytical factors have not kept pace and continue to cause bottlenecks in this regard. For example, Yang and colleagues (2013) note the need to improve the run-time of error correction algorithms and validation procedures involving hybrid datasets generated by multiple platforms [38]. However, while fast turnaround will be important for future applications of epigenomics, transcriptomics, or proteomics in diagnostic contexts, where profiles are dynamic and there is a need for rapid assessment of a person’s current status, the genome effectively remains unchanged over time and can therefore be determined in advance (“pre-emptively”) before health problems arise, making turnaround time essentially irrelevant. So, in the context of pharmacogenomics, speed may be a relatively unimportant criterion for pre-emptive testing, although it remains an issue if rapid clinical decision making is required for a patient who has not been previously genotyped.

Infrastructure also requires consideration. Ideally, it should ultimately be possible to perform genotyping and data interrogation in close proximity to the point-of-care e.g., within a hospital laboratory or in community settings such as a pharmacy or GP office. Equipment for running the assay should, therefore, be user-friendly and self-contained, while data analysis and reporting should be compatible with the computing power provided by a standard desktop or laptop computer. Technologies such as Oxford MinION, while still evolving, hold considerable promise in this context [57].

Data storage and linkage are among the most important limiting factors in many clinical contexts. For example, the relevant data generated by a platform should be sufficiently compact to link with electronic health records. Given that current microarray platforms can screen approximately 500,000–1 million or more SNPs or 1 million probes for detecting copy number variation for a few hundred dollars, and that the resulting SNP data can be compressed to a few megabytes, this technology would appear the most appropriate fit based on the above criteria [58]. The need for infrastructure that can handle “big data” is another potential limitation of NGS that makes it currently less feasible for clinical applications. In terms of data storage, the raw compressed fastq files from a single whole genome sequencing run at 30× sequencing depth amount to ~100 gigabytes; on top of this, the aligned, processed files require storage of around 1–1.5 terabytes per patient [59]. While advances in cloud storage and data transmission technologies will help overcome problems associated with storing these massive volumes of data, at least with regard to pharmacogenomics, it is debatable whether this is an economically intelligent use of resources given the small number of known variants that modify drug responses.

## 5. Ethical, Legal, and Social Issues (ELSI)

Several other issues also need to be taken into account in addition to the above considerations, most notably ELSI. In this regard there are a range of potential concerns, including privacy and unanticipated results. Irrespective of the technology, some of these concerns can be addressed by appropriate consent procedures such as “traffic light” systems similar to those being used in related contexts, such as consent for the release of an individual’s genomic information for research purposes [60]. Such systems can be used to enable people to give different levels of consent for different categories of genomic information. For example, with respect to privacy, a person might elect not to provide certain kinds of genomic information for commercial purposes but may be happy to provide this information for other research purposes. With respect to unanticipated results, a person might elect not to be informed about a mutation predisposing to an untreatable condition. However, it is worth noting that, irrespective of the technology used (microarray or NGS), these issues are far less likely to present concerns for pharmacogenomic tests than for genomic diagnostic testing. The presence of a pharmacogenomically actionable mutation is generally innocuous unless a person takes a medicine affected by the mutation, in which case the information becomes potentially advantageous, reducing the likelihood of an adverse reaction or therapeutic failure.

Another important ethical concern surrounding pharmacogenomics relates to justice and equity. As pharmacogenomics and precision medicine begin to assume pivotal roles in healthcare, there will be increasing need to ensure fair, even distribution of benefits to prevent further widening of the gaps that exist between individuals of different socioeconomic status and, in particular, people from developing or resource-limited countries, who may already be disadvantaged with respect to healthcare. Such countries are also least able to sustain any inefficiencies in healthcare systems arising as a result of drugs that were originally developed for use in other populations not working appropriately because of genomic differences.

Countries from Europe, Africa, Asia, and the Pacific have now initiated the implementation of genomic approaches in health care. In view of the foregoing discussion and taking into consideration the issues of inadequate funds, lack of access to technology, and scarcity of well-trained health experts in developing or resource-limited countries, currently the only viable approach to ensure that these countries are given equitable opportunities to benefit from these new initiatives would appear to be the utilization of microarray techniques, rather than next-generation approaches. Initiatives implementing large-scale pharmacogenomics are now starting to appear worldwide. For example, the European Ubiquitous Pharmacogenomics network [61] project provides information on the prevalence and effects of pharmacogenomically relevant gene variants in Europe with particular focus on developing countries, in order to generate locally-relevant drug dose recommendations [62]. Pharmacogenomics networks are also being set up in Asia, for example the Asian Network for Pharmacogenomics [63]. The Human Heredity and Health in African (H3Africa) initiative, backed by the US NIH, the UK Wellcome Trust, and the African Society of Human Genetics, aims to develop the capacity of African scientists to apply genomic and epidemiological approaches in locally-relevant clinical contexts [62,64].

## 6. Using Microarrays for Pre-Emptive Pharmacogenomics Testing

One example of successful array-based pharmacogenomics in practice is the Pharmacogenomic Resource for Enhanced Decisions in Care and Treatment (PREDICT) program at Vanderbilt University Medical Centre [54]. The PREDICT program uses panel-based genotyping to identify specific SNPs that are known to have drug-response associations, allowing tailored clinical decision support to be provided for each participant. In an initial study of almost 10,000 participants, which focused on only five well-established drug-gene interactions (clopidogrel—CYP2C19; simvastatin—SLCOB1; warfarin—CYP2C9 and VKORC1; thiopurines—TPMT; tacrolimus—CYP3A5), one or more actionable variants were identified in 91% of genotyped participants [54]. The clinical utility of this approach is, therefore, likely to be considerably greater than single gene tests. In addition, the pre-emptive, panel-based genotyping approach used in this study enabled substantial reduction in the testing burden compared to single gene assays and facilitated the provision of results at the point of care. This study testifies that custom-designed arrays are appropriate for accurate identification of common SNPs in accredited, quality-controlled pharmacogenomic screening services.

As was also noted in the introduction, a recent review has highlighted the success of array-based pre-emptive pharmacogenomics testing in several other US medical centers, namely St Jude’s Children’s Research Hospital, University of Florida and Shands Hospital, the Mayo Clinic, and Mount Sinai Medical Centre [65]. While the genotyping platform varied among the centres, as did the number of genes assayed (ranging from 34 to 230), each program identified a high prevalence of actionable variants. In fact, when considering only 12 pharmacogenes, it is estimated that over 97% of the population of the US have at least one actionable high-risk diplotype [65]. The experience of these trailblazing centres in instituting pre-emptive pharmacogenomics testing has already highlighted challenges and solutions to implementation, paving the way for smooth deployment in other locations.

## 7. Conclusions

Advances in sequencing technologies have revolutionised genomic discovery in the lab, while concomitant reductions in cost will increase the feasibility of employing such technologies in routine clinical or pharmacy practice. Yet the availability of new technologies should not dictate that its predecessors be discarded for every application. In the context of widespread pharmacogenomics profiling of large numbers of individuals, existing microarray technology offers considerable advantages over sequencing with respect to cost of infrastructure, ease of analysis, interpretation, and logistics of data storage and interrogation. In contrast, NGS offers no obvious advantages over array-based methods for screening large numbers of common variants. While the urge to embrace an exciting new technology as a panacea can sometimes seem irresistible, we hope that common sense will prevail in judging the utility of genomics technologies in a context-dependent manner.

## Figures and Tables

**Table 1:** Genes considered to have high levels of evidence for effects on drug responses according to PharmGKB and CPIC Gene and Drug Guidelines.

Genes	Drugs	CPIC	PharmGKB	CPIC Publications
*HLA-B*	Abacavir; allopurinol; phenytoin; carbamazepine	A	1A	[14,15,16,17]
*CYP2C19*	Amitriptyline; clopidogrel; imipramine *; trimipramine *; citalopram; escitalopram	A	1A	[18,19,20]
*CYP2D6*	Amitriptyline; codeine; desipramine; doxepin; fluvoxamine; imipramine; nortriptyline; paroxetine; trimipramine	A	1A	[18,20,21]
*UGT1A1*	Atazanavir	A	1A	[22]
*TPMT*	Azathioprine; mercaptopurine; thioguanine	A	1A	[23,24]
*DPYD*	Capecitabine;fluorouracil; tegafur	A	1A	[25]
*CFTR*	Ivacaftor	A	1A	[26]
*CYP2C9*	Warfarin; phenytoin **	A	1A	[16,27]
*G6PD*	Rasburicase	A	1A	[28]
*SLCO1B1*	Simvastatin	A	1A	[29,30]
*CYP3A5*	Tacrolimus	A	1A	[31]
*VKORC1*	Warfarin	A	1A	[27]
*IFNL3*	Peginterferon alfa-2a; peginterferon alfa-2b; ribavirin; telaprevir	A	1A	[32]
*CYP2B6*	Efavirenz	B	1B	
*CYP4F2*	Warfarin	B	1B	
*ANKK1*	Bupropion	D	1B	
*GRIK4*	Citalopram	D	1B	

* PharmGKB level of evidence 2A; ** PharmGKB level of evidence 1B.
